# The dynamics of statistical learning in autism – exploratory research

**DOI:** 10.1192/j.eurpsy.2024.440

**Published:** 2024-08-27

**Authors:** C. A. Nagy, F. Hann, B. Brezóczki, K. Farkas, T. Vékony, O. Pesthy, D. Nemeth

**Affiliations:** ^1^Institute of Psychology, Eötvös Loránd University; ^2^Doctoral School of Psychology, Eötvös Loránd University; ^3^Brain, Memory and Language Research Group, Institute of Cognitive Neuroscience and Psychology, Research Centre for Natural Sciences; ^4^Department of Psychiatry and Psychotherapy, Semmelweis University, Budapest, Hungary; ^5^INSERM, Université Claude Bernard Lyon 1, CNRS, Centre de Recherche en Neurosciences de Lyon CRNL, Bron, France

## Abstract

**Introduction:**

In the context of developmental disorders, it is frequently observed that atypical processes may yield seemingly unimpaired behavioural outcomes. Research has shown that children and adults with Autistic Spectrum Disorder (ASD) have intact statistical learning performance. Recent studies have indicated that learning can happen not only during practice but during ultrashort rests between practice blocks (that is, ultrafast offline learning) but no study to date examined these dynamics in ASD.

**Objectives:**

This research aimed to unravel the effect of ASD on learning during and between blocks, also known as online and offline improvement.

**Methods:**

We conducted a series of research with three different samples: 1) ASD children (N = 27), 2) ASD adults (N = 42), and 3) neurotypical adults with distinct positions on the autism spectrum, i.e., the severity of autistic traits (N = 174). Participants performed the Alternating Serial Reaction Time task, allowing us to measure statistical learning (the extraction of statistical knowledge) and general skill learning (speed-up regardless of probabilities) separately.

**Results:**

Individual differences in online and offline improvements were observed. Results of individual studies further confirmed by meta-analysis performed on the three above-mentioned datasets show that neither ASD nor the severity of autistic traits influences the dynamics of learning.

**Conclusions:**

Our findings suggest that, not only learning but also the dynamics of acquisition of statistical knowledge are intact in autism.

**Disclosure of Interest:**

None Declared

